# The delayed arrival of faster solar energetic particles as a probe into the shock acceleration process

**DOI:** 10.1093/nsr/nwaf348

**Published:** 2025-08-26

**Authors:** Yuncong Li, Jingnan Guo, Daniel Pacheco, Yuming Wang, Manuela Temmer, Zheyi Ding, Robert F Wimmer-Schweingruber

**Affiliations:** National Key Laboratory of Deep Space Exploration/School of Earth and Space Sciences, University of Science and Technology of China, Hefei 230026, China; Institute of Physics, University of Graz, Graz 8010, Austria; National Key Laboratory of Deep Space Exploration/School of Earth and Space Sciences, University of Science and Technology of China, Hefei 230026, China; CAS Center for Excellence in Comparative Planetology, University of Science and Technology of China, Hefei 230026, China; National Key Laboratory of Deep Space Exploration/School of Earth and Space Sciences, University of Science and Technology of China, Hefei 230026, China; National Key Laboratory of Deep Space Exploration/School of Earth and Space Sciences, University of Science and Technology of China, Hefei 230026, China; CAS Center for Excellence in Comparative Planetology, University of Science and Technology of China, Hefei 230026, China; Institute of Physics, University of Graz, Graz 8010, Austria; Institute of Experimental and Applied Physics, Kiel University, Kiel DE24118, Germany; Institute of Experimental and Applied Physics, Kiel University, Kiel DE24118, Germany

**Keywords:** solar energetic particle, coronal mass ejection shock, particle acceleration, magnetic connectivity

## Abstract

The particle acceleration and transport process during solar eruptions is a critical and long-standing problem in space plasma physics. Through decades of research, it is well accepted that particles with higher energies released during a solar eruption arrive at observers earlier than the particles with lower energies, forming a well-known structure in the dynamic energy spectrum called particle velocity dispersion, as frequently observed by space missions. However, this picture is challenged by new observations from NASA’s Parker Solar Probe and ESA’s Solar Orbiter that show an unexpected inverse velocity dispersion (IVD) phenomenon, where particles with higher energies arrive later at the observer. Facing this challenge, here we report the recent observations of such IVD structures with 10 solar energetic proton events observed by Solar Orbiter, and then analyze the mechanisms causing this unusual phenomenon. We suggest that shock diffusive acceleration, with respect to magnetic reconnection, is probably a dominant mechanism to accelerate protons to tens of mega-electron-volts in such events where particles need longer time to reach higher energies. Furthermore, we determine, innovatively, the physical conditions and time scales during the actual shock acceleration process that cannot be observed directly.

## Teaser

A new type of solar energetic particle events with faster particles arriving later than slower ones has been recently observed by spacecraft at a solar distance smaller than 1 astronomical unit. This new feature helps us better understand how shocks accelerate particles and what conditions determine the energy and release of particles into the interplanetary space.

## INTRODUCTION

Solar energetic particles (SEPs) are accelerated by flares and/or shocks driven by coronal mass ejections (CMEs) [1,2]. SEPs constitute an important radiation hazard for robotic and crewed space missions and having an understanding of them is of capital importance to ensure the success of future space explorations [3]. Previous observations often showed that more energetic (and therefore faster) particles arrived earlier than less energetic particles during solar eruptions [4–6]. This supports the idea that the acceleration time scale is relatively short so that the accelerated particles are released at approximately the same time irrespective of their energy and propagate along similar trajectories. This phenomenon is called particle velocity dispersion (VD; see, e.g. [7–9]).

Since the launch of Solar Orbiter (SolO) in 2020 [10], a detailed dataset of remote and *in situ* observations of solar eruptions have been collected. In particular, the unprecedented resolution of energetic particle observations has opened a new window looking into the fine structures of SEP events and dynamics [7,11–13]. As solar activity has been rising as the Sun enters the solar maximum of cycle 25, SolO has observed an increasing number of events, some of which defy our previous understanding of VD. During these unusual events we observe the known VD feature for particles below an event-specific energy, but particles at higher energies appear to arrive later with increasing energy. This behaviour has been referred as ‘inverse velocity dispersion’ (IVD) based on a single case observed by the Parker Solar Probe (PSP) when it was very close to the Sun (at around 0.07 au; [14,15]). Up to the end of 2024, SolO had observed at least 10 such events for distances from the Sun between 0.49 and 0.95 au, and for a wide range of relative longitudinal separations between the solar source and SolO.

In this paper we present the 10 events observed so far and analyse three especially clear examples in more detail. We find that the IVD features can be well interpreted by the diffusive shock acceleration (DSA) mechanism through which particles need a longer time to obtain higher energies before being released from the acceleration site. This finding suggests that DSA is probably a dominant mechanism in such events, with respect to magnetic reconnection, to accelerate protons to tens of mega-electron-volts that could be potentially radiation damaging to instruments and humans in space under thin shielding. We further determine, innovatively, the physical conditions and time scales during the actual acceleration process of these particles at the shock that cannot be observed directly.

## RESULTS

Since early 2022 to mid-2024, SolO has observed many SEP events, among which we select those with IVD features using the following criteria.

1. The event must have a clean background, unaffected by the preceding events at the main energy range covered by SolO’s energetic particle detector (EPD; [16,17]).
2. The proton increase must show clear VD and IVD parts, with a transition of the two structures from low to high energies.
3. SolO must be in front of the solar disk as seen from Earth so that Earth-based remote-sensing observations can be useful (see the Dataset section within the online supplementary material).
4. White-light coronagraphs onboard the Solar-Terrestrial Relations Observatory Ahead (STEREO A; [18]) and the Solar and Heliospheric Observatory (SOHO; [19]) should observe the CME simultaneously so that the original direction and speed of the CME can be derived (see the Dataset section within the online supplementary material).

A total of 10 IVD events were selected based on the first two criteria, with only three events satisfying all four conditions and being analysed in detail. We first present a detailed analysis of the 9 November 2023 event because it had the clearest association of flare, CME and SEPs. Together with another two events (24 December 2023 and 31 December 2023), the results are summarized in Table 1. Further information for all 10 IVD events is given in Table 2 and the online supplementary material.

**Table 1.** Observations and analysis results of three different SEP events with clear IVDA features and their associated solar eruptions.

	Event dates		
	9 November 2023	24 December 2023	31 December 2023
**Observation**			
Distance of SolO	0.66 au	0.94 au	0.95 au
Flare location	W04, S10	W29, S19	W89,N09
Flare class	C2.6	M2.6	C2.8
$\Delta \phi$	21	7.2	-46.4
Flare start and end times (UT)	10:45–11:29	16:29–16:48	11:47–12:17
Flare peak time (UT)	11:05	16:41	11:59
CME eruption time^a^ (UT)	11:40	15:40	9:52
CME starting speed	$870\pm 87$ km/s	$740\pm 74$ km/s	$830\pm 83$ km/s
Time of type-II radio burst (UT)	11:05–14:16	14:51–17:28	10:47–12:04
$E_{0}$ (first arrival energy)	3 MeV	1-4 MeV	2 MeV
SEP onset	13:32	23:09	15:15
$E_{m}$ (maximum energy observed by SolO)	50 MeV	12 MeV	—^b^
$V_{SW}$ ^c^	$350\pm 70$ km/s	$300\pm 60$ km/s	$400\pm 80$ km/s
$\gamma _{{VD}}$ (spectral index of the VD particles)	-2.14	-1.94	-0.61
$\gamma _{{IVD}}$ (spectral index of the IVD particles)	-3.41	-3	-3.66
**VD and IVD analyses**			
$t_{0}$ (UT; derived from VDA)	11:39 $\pm$ 7 min	13:44 - 16:35	12:04 $\pm$ 6 min
Path length (derived from VDA)	$0.89 \pm 0.21$ au	$1.61 \pm 0.45$ au	$1.33 \pm 0.17$ au
Parker spiral length	0.72 au	1.04 au	1.09 au
Releasing radial distance^d^	0.05–0.16 au	0.14–0.38 au	0.07–0.2 au
Slope of $E^{\prime }$ vs. $t_{\rm release}({E}^\prime)$	9.25 MeV/h	2.88 MeV/h	0.69 MeV/h
**Shock parameters**			
$u_{u}$ (shock upstream speed)	-542 km/s	-440 km/s	-430 km/s
Derived $u_{d}$ (shock downstream speed)	-358 km/s	-275 km/s	-291 km/s
*r* (shock compression ratio)	1.5	1.6	1.5
$\theta$ (shock normal angle)	20	25	—
$\lambda _{0}$ at $t_{\rm release}$ derived from IVDA	$1.2\times 10^{-4}$ au	$4.5\times 10^{-4}$ au	$2.5 \times 10^{-4}$ au
*In situ* upstream $\lambda _{\parallel }^{\rm e}$	$(0.8\\!-\\!2)\times 10^{-2}$ au	$(2\\!-\\!6) \times 10^{-3}$ au	—
The derived radial dependence of	$D^{1.6}$	$D^{0.8}$	—
$\lambda _{\parallel }$ on solar distance *D*			

$^{\rm a}$ First appearance in C2. The times for the flares and CMEs are the observation times minus the time for the light to travel from the Sun to SolO. $^{\rm b}$The second SEP event captured by SolO contaminated the IVD structure of the current event. $^{\rm c}$*In situ* solar wind speed averaged over 10 h before the shock arrival. $^{\rm d}$The modelled distance of the shock upon the derived IVD particles’ release time. $^{\rm e}$The parallel mean free path derived from the e-folding time method.

**Table 2.** Summary of 10 SEP events with IVD features. The SEP observations by SolO and spacecraft connectivities for each event can be found in the online supplementary material.

	Start date	R$_{\text{Sun-SolO}}$	Onset *E*	Max. *E*	Onset	IVD	$\delta _{\text{SolO-source}}$	$\delta _{\text{footpoint-source}}$
No.	(yyyy-mm-dd)	(au)	(MeV)	(MeV)	(UT)	duration (h)	(deg)	(deg)
1	2023-11-09	0.66	3	10	13:32	4	$-16$	21
2	2023-12-24	0.94	1–4	12	23:09	12	$-42$	10
3	2023-12-31	0.95	2		15:15	8	$-102$	52
4	2022-03-10	0.46	7	70	21:00	1	$-67$	$-31$
5	2022-06-07	0.96	0.7	20	13:00	12	$-13$	52
6	2022-06-26	1.01	1	10	10:30	15	13	82
7	2022-07-23	0.99	2–10	50	22:30	8	$-24$	44
8	2023-01-20^a^	0.95	0.6	7	23:30	13	$-34$	24
9	2023-08-07	0.88	3	60		8	83	133
10	2024-03-23	0.39	20		01:30	4	23	42

$^{\rm a}$ Contaminated by a subsequent event before it ends.

### Overview of the 9 November 2023 event

**Figure 1.**
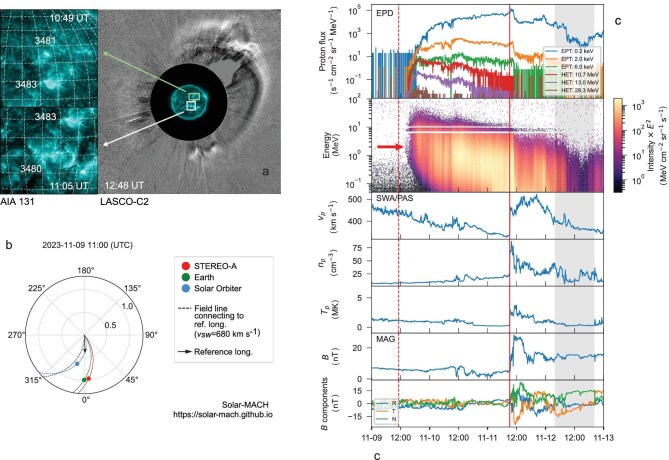
Overview of the solar eruption on 9 November 2023 and *in situ* observations of the SEP event and interplanetary environment. The right image of (a) shows the EUV (131 Å) observation of the Sun and white-light coronagraph observation of the CMEs from Earth’s view. The left image of (a) shows an enlarged view of the two candidate source regions (flares). Panel (b) shows the flare direction, positions and magnetic connectivity of SolO and other spacecrafts in the solar ecliptic plane. Panel (c) shows the *in situ* measurements at SolO including, from top to bottom, the energetic proton flux for six different energy bins (shown in the legend), energetic proton dynamic spectrum across the whole energy range from $\sim$50 keV to 105 MeV, solar wind bulk speed, proton density, proton temperature, magnetic field magnitude and the magnetic field vector components in Radial–Tangential–Normal (RTN) coordinates. The red dotted line marks the peak of the flare shown by the SolO hard X-ray observations (see also the online supplementary material and Table 1); the red solid line indicates the shock arrival at SolO; the grey-shaded area marks the duration of the CME that drove the shock. All particle measurements were obtained from EPD’s sunward-looking telescopes.

Using solar observations (see the Dataset section within the online supplementary material), the 9 November 2023 SEP event could be associated with two C-class flares (C1.3 observed between 10:41 and 11:07 UT from AR 13481 located at N24W15 and C2.6 observed between 10:53 and 11:37 UT from AR13480 located at S10W04) accompanied by a wide halo CME as seen from Earth (Fig. 1a). The extreme ultraviolet (EUV) observation of the Sun (in 193 Å) shows the two flares and their enlarged images (in 131 Å). The CME shock can be clearly seen in the running difference image of the white-light coronagraph. A movie of this time period showing the flare and CME eruption can be found at https://cdaw.gsfc.nasa.gov/movie/make_javamovie.php?date=20231109&img1=sdo_a193&img2=lasc2rdf, which indicates that there may be a prior CME heading north that interacted with the halo CME, with the latter most likely related to the C2.6 flare located in the southern hemisphere. The speed of the halo CME-driven shock is derived to be 870 km/s based on quasi-graduated cylindrical shell (GCS; [20]) fits when the shock was clearly shown in the coronagraph.

The CME speed and flare classes were not among the highest on record; nevertheless, clear solar radio bursts were observed that are associated with the shock propagation and particle release process. Type-III emission was observed at the beginning of the flare eruption and a slow drift of the type-II radio burst started from $\sim$1 MHz to 20 kHz (see the online supplementary material).

Figure 1b shows the position of SolO [21], which was at a solar distance of 0.66 au, and other spacecrafts in the solar ecliptic plane during the event. The coloured Parker spiral lines approximating the direction of the interplanetary magnetic field (IMF) from the Sun to different observers are plotted based on the measured solar wind speed (which was about 430 km/s at SolO around the SEP onset time; 680 and 700 km/s at Earth and STEREO A, respectively). The arrow shows the longitude of the C2.6 flare that approximates the associated CME eruption and its propagation direction. The dashed line represents the best magnetic connectivity to the flare based on Earth-observed solar wind speed.

**Figure 2.**
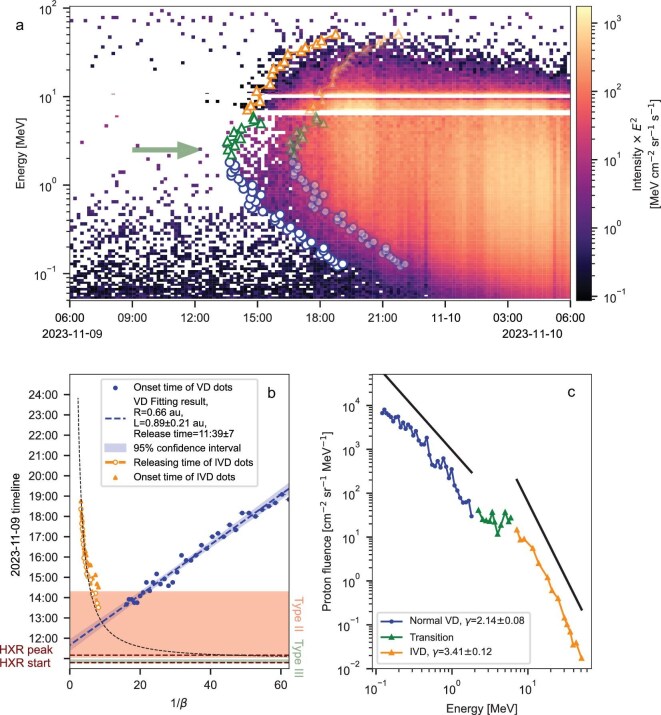
(a) Dynamic spectra of the early phase of the 9 November 2023 event. (b) The release time and path length derived from the VDA of protons below $\sim$2 MeV and from the IVD release time analysis of protons above $\sim$7 MeV. (c) The 3-h-integrated proton energy spectra starting from the onset of each energy range (between the two sets of markers in (a)). In (a), the onset times for VD particles are marked with blue circles, while the onset times for IVD particles are indicated with orange triangles (see the Methods section below for onset time determination). The big green arrow indicates the first arrival energy within the transition range between the VD and IVD energies, marked with green triangles. The onset time is used in panel (b) to derive the release time and path length of energetic protons shown in the legend (see Equations (7) and (8) in the Methods section). The theory-fitted release time based on DSA theory is plotted as the dotted line. The timings of the C2.6 flare hard X-ray ($\sim$10 keV) are marked with horizontal dashed lines; type-II and type-III radio bursts shifted to the solar surface (subtracting 8.33 min, accounting for the time that photons need to reach Earth) are shown as pink and green bands, respectively. In (c), the power-law fitting obtained for the low- and high-energy parts is marked by the black lines.

Figure 1c shows combined *in situ* measurements at SolO, including energetic particles, solar wind plasma and the IMF. The first panel presents the intensity-time profile for protons at different energy bins. The second panel shows the dynamic spectrum of proton flux covering a continuous energy range from $\sim$50 keV up to 105 MeV. The SEP event starts at 13:32 UT on 9 November 2023, which is the onset time of the first arrival energy, indicated by the red arrow at around 3 MeV. This plot shows a clear IVD component at energies above a few mega-electron-volts in addition to the normal VD part at lower energies. A more detailed analysis of SEP transport based on the two components is shown in Fig. 2 and discussed later.

Figure 1c shows that at the onset of the SEP event, most plasma and magnetic conditions were in a relatively quiet state. About two days later, the *in situ* plasma information provides the signature of a shock arrival, as indicated by the red solid line. Considering the timing and speed of the shock, as also supported by the EUHFORIA simulation (available in the online supplementary material), this should have been the shock associated with the SEP event. Upon the shock arrival, energetic storm particles (ESPs), which are trapped near the shock by self-generated waves [22], were also seen in energy channels below a few mega-electron-volts. In the sheath and within the CME, fine structures of the SEP flux are nicely seen that may be related to rapid changes of magnetic structures in the solar wind [7].

### SEP release time and path length

Figure 2 illustrates the typical analysis performed to obtain the release time and path length derived from the velocity dispersion analysis (VDA; [23]) of protons under $\sim$2 MeV as well as the release time of IVD particles with different energies above $\sim$7 MeV. VD and IVD analyses follow the methods described in the Methods section below. The dynamic spectrum in panel (a) shows a triangular structure with low-energy VD component and high-energy IVD part for the 9 November 2023 event. The transition energy is around 3 MeV (marked by the green triangles between 2 and 7 MeV), i.e. these are the particles arriving earliest at SolO and have very low counting statistics. Besides, it is not clear which component (VD or IVD) they belong to. For this reason, we have ruled out particles in this energy range in the following study.

The VDA results are shown in Fig. 2b as the blue points and the linear fit, with the release time $t_{\text{0}}$ derived as 11:39$\pm$7 UT, which is broadly consistent with the hard X-ray (HXR) peak time and the start of type-II radio emission (both were 11:05 UT). This indicates that these particles are possibly accelerated during the flare reconnection process and/or the initial phase of the shock when it was still in the low corona. Notably, the VDA-derived path length is longer than the corresponding Parker spiral length (also for the other two events shown in the online supplementary material and Table 1). This supports the view that energetic particles may experience transport effects such as scattering or cross-field propagation[22,24] and also that the Parker spiral approximation is an over-simplified situation that has been challenged by recent studies [4,25].

The IVD structure indicates that these particles were not released at the same time and that particles with higher energies were released later, a scenario more likely related to shock acceleration rather than flare acceleration processes (with further explanations provided in the Discussion section below). We derive their energy-dependent release time, starting from an initial guess that they followed the same path length as the VD particles. This path length is further iterated, taking into account the shock propagating away from the Sun, thus reducing the particle paths to the observer (see the Methods section below and Equation (8) therein for further details). The derived release time $t_{\text{release}}(E^{\prime })$ for each energy $E^{\prime }$ is marked in Fig. 2b with orange circles that clearly show that higher-energy protons (to the left of the *x* axis) are released later than lower-energy ones.

If VD and IVD particles do come from different sources and different acceleration processes, their energy spectra may be different. In order to resolve the energy spectrum most similar to that upon the release, we use particle flux during the initial three hours following the onset of each energy range featuring particles that experienced the least scattering [26]. We then deduce the energy spectra of VD and IVD particles, respectively, and find that they show different power-law indices (Fig. 2c). For reference, two black lines are shown with power-law indices of 2.14 and 3.41 for low-energy VD and high-energy IVD parts, respectively.

SEP spectra often show double power-law features, but their origin is still under debate, possibly due to the transport effect [27,28] or as a direct consequence of the time-dependent shock acceleration process, which can reflect properties of the shock geometry [29–31]. We suggest that, in this event, the double power-law feature indicates that there may be two different acceleration phases, e.g. DSA for the high-energy part, as discussed in detail later, and flare reconnection or early-stage-shock acceleration for the low-energy part, consistent with the results indicated by the release time analysis in Fig. 2b.

## DISCUSSION

Interplanetary shocks are the main source of high-energy protons in the inner heliosphere causing the commonly known gradual SEP events [1]. Assuming that the IVD particles are mainly accelerated at the shock while it propagates outwards, we interpret the origin of the IVD feature by exploring two different scenarios (which may also be co-responsibly contributing agents).

### Acceleration time evolution during diffusive shock acceleration

Among various particle acceleration mechanisms in the heliosphere, DSA is one of the most common and important mechanisms [32,33], especially for parallel and quasi-parallel shocks. DSA can be explained through the Fermi acceleration process, during which particles could cross the shock multiple times by elastically scattering off magnetic irregularities that converge at the shock. Particles gain a small amount of energy in each traverse of the shock front. Strong turbulent magnetic fields help the shock to trap particles, which can be continuously accelerated, while the final energy of the escaping particle is determined by its initial energy and the duration time of the acceleration as well as the shock properties [32]. Bell [34] and Drury [33] developed an individual particle approach to compute the energy (or momentum) gain of a particle from one crossing through the shock, and Jones [35] proposed a simpler way to obtain this value, just combining the equation for the conservation of particles,


(1) \begin{eqnarray*}
\frac{\partial }{\partial x}(j_x)+\frac{\partial }{\partial p}(j_p)=0,
\end{eqnarray*}

where $j_x$ and $j_p$ are the fluxes of particles along the *x* (shock normal direction) and *p* (particle momentum) axes, respectively, with the diffusion-convection equation [36]


(2) \begin{eqnarray*}
&&\frac{\partial }{\partial x}\bigg [uf(x,p)-\kappa \frac{\partial f(x,p)}{\partial x}\bigg ]\nonumber\\
&&\quad \qquad =\frac{1}{3}\bigg (\frac{\partial u}{\partial x}\bigg )\frac{\partial }{\partial p}[pf(x,p)],
\end{eqnarray*}

which leads to the final average momentum of a particle with initial momentum $p_0$ after crossing the shock *N* times:


(3) \begin{eqnarray*}
\langle p\rangle (N)=\prod _{i=1}^N\bigg [1+\frac{2}{3}(u_u-u_d)/v_i\bigg ]p_0,
\end{eqnarray*}

with $u_u$ ($u_d$) the upstream (downstream) flow speed in the shock frame and $v_i$ the particle speed.

Based on the magnetic field data across the shock front when it arrived at SolO (see the solid vertical line in Fig. 1c) on 9 November 2023, we show that the angle between the shock normal and upstream magnetic field $\theta _{Bn}$ is about $20^{\circ }$, i.e. the shock qualifies as a quasi-parallel shock, indicating that DSA may likely have been the main acceleration mechanism during this long-lasting SEP event, if the shock direction did not evolve significantly over the distance.

There is no easy way to directly resolve the shock properties for accelerating first-arriving IVD particles when the shock was closer to the Sun. Here we use the accelerated IVD particle spectral index (Fig. 2c) to derive the downstream speed when it was accelerating the IVD particles based on the method derived by Bell [34] and Jones [35]. In detail, they considered the probability of particles crossing the shock *N* times and used this to derive the particle distribution and spectrum. Assuming an isotropic particle distribution in the local plasma flow frame, one can calculate the probability that a particle, having once crossed the shock in the configuration space, will return. Additionally, the probability that a particle returns at least N/2 times can be determined, which is equivalent to the probability of the particle crossing the shock at least *N* times.

The density function can be given by the partial differentiation of the probability function with respect to the momentum:


(4) \begin{eqnarray*}
f(p)=\bigg (\frac{n_0}{p_0}\bigg )\bigg (\frac{3r}{r-1}\bigg ) \bigg (\frac{p}{p_0}\bigg )^{-\sigma }.
\end{eqnarray*}

Here $p_0$ and *p* are the initial and final momentums of the particle (same as defined before), $r\equiv \rho _d/\rho _u=u_{u}/u_{d}$ is the compression ratio of the shock, $\sigma \equiv (r+2)/(r-1)$ and $n_0$ is the upstream number density. Equation (4) presents the well-known form of the accelerated particle energy spectrum, exhibiting a power-law behaviour of $dJ/dE \propto E^{-\sigma /2}=E^{-\Gamma }$. Here, the compression ratio *r* is constrained to $1<r<(\gamma _G+1)/(\gamma _G-1)$ for a non-relativistic monoatomic gas, where $\gamma _G=5/3$. Consequently, the upper limit of *r* is 4 so that the energy spectral index $-\Gamma$ should be larger than 1.

Based on the derived particle spectral index $-\Gamma$ and the measured upstream flow speed $u_u$ (in the shock frame), we can determine the compression ratio *r*, and, subsequently, the downstream flow speed can be given as


(5) \begin{eqnarray*}
u_d=\frac{(2\Gamma -1)u_u}{2+2\Gamma }.
\end{eqnarray*}

Applying the method to the 9 November 2023 event, the *in situ* measured upstream flow speed is $u_u=-542$ km/s (perpendicular to the shock surface and in the shock frame) upon the shock arrival. The spectral index of the IVD onset particles is 3.41, which corresponds to a shock compression ratio of $r=1.5$ in DSA theory. We then derive the downstream flow speed $u_d=-358$ km/s when the shock was close to the Sun. In the above calculation, we have assumed that the shock properties and the seed particle spectra do not change over the distance from about 0.05 to 0.14 au. Future investigations can consider the evolution of shock properties and the seed particles so that the IVD spectrum actually combines injection sources at different times.

Two additional events on 24 December 2023 and 31 December 2023 also have clear IVD features with sufficient remote-sensing data support. We also studied them, with the results shown in Table 1.

**Figure 3.**
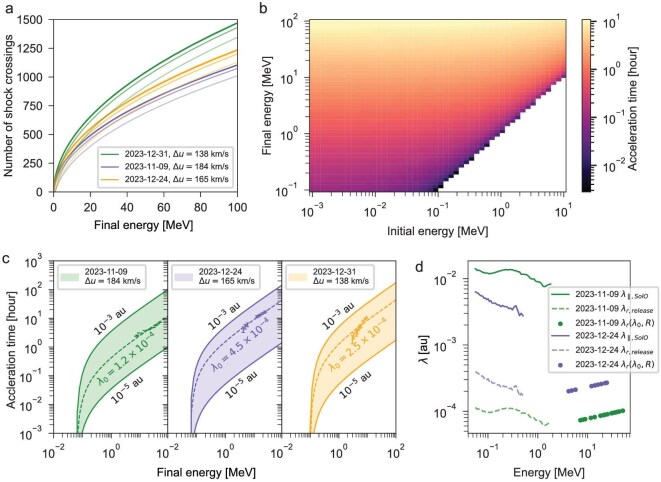
(a) Shock crossing times versus the final proton energy derived from different events with different shock properties (shown as different $\Delta u$ in the legend). For each event, four lines transitioning from darker to lighter represent an increase in the initial kinetic energy: 10$^{-3}$, 10$^{-2}$, 10$^{-1}$ and 1 MeV. (b) Acceleration time matrix for the 9 November 2023 event. Colour scale represents the acceleration time $\tau _a$ required to accelerate a particle from an initial energy (*x* axis) to a final energy (*y* axis), with Equation (6) using $\lambda _0=1.2 \times 10^{-4}$ au. The matrices for other events and other $\lambda _0$ values have similar structures and are not shown here. (c) The acceleration time $\tau _a$ versus the final proton energy for three different events. Each coloured band corresponds to the possible solutions of $\tau _a$ with $\lambda _0$ ranging from $10^{-5}$ to $10^{-3}$ au. The markers with error bars are results derived from the observations based on IVD analysis. (d) The upper two lines are the parallel mean free path $\lambda _\parallel$ derived from the ESP flux when the shock arrived at SolO for the 9 November 2023 and 24 December 2023 events (while the 31 December 2023 event had a contamination by a following SEP event). The lower dashed lines are the $\lambda _{r, {\rm release}}$ at the acceleration source region derived from the upper ones considering the radial gradient of $\lambda _\parallel$. The value of $\lambda _\parallel$ obtained from fitting the observations in panel (c) is plotted (above $\sim$7 MeV as dots) as a reference. See the text for further details.

The number of times a particle needs to traverse the shock front, *N*, to accelerate from its initial momentum $p_0$ to a specified momentum *p* is obtained via Equation (3). It also shows that the difference between the upstream and downstream shock speeds, defined as $\triangle u = \vert u_u-u_d\vert$, can influence the energy gain: larger $\triangle u$ would result in more efficient acceleration. Figure 3a shows the required number of crossings for protons reaching different energies with the $\triangle u$ derived from the 9 November 2023 event and another two selected events. For each event, lines that transition from darker to lighter colours represent an increase in the initial $p_0$.

A more quantified acceleration time scale can be obtained by transiting from a single-particle approach to a macroscopic perspective. Drury [33] first derived the mean acceleration time from the steady, time-dependent solution of the transport equation, under the condition that a mono-energetic source of particles $Q\delta (p-p_0)$ is available at $t=0$, and the distribution of accelerated particles is $f(t,x,p)=0$ at $t=0$. The expression for the mean acceleration time is derived as


(6) \begin{eqnarray*}
\tau _a = \frac{3}{u_u - u_d} \int _{p_{\text{inj}}}^{p} \kappa _{rr} \bigg ( \frac{1}{u_u} + \frac{1}{u_d} \bigg ) \frac{dp^{\prime }}{p^{\prime }},
\end{eqnarray*}

where $p_{\text{inj}}$ is the initial momentum of the source particles, and $\kappa _{rr}$ is the effective diffusion coefficient along the shock normal in both upstream and downstream regions [37] related to the radial mean free path $\lambda _{rr}$, given by


\begin{eqnarray*}
\kappa _{rr}=\frac{v}{3} \lambda _{rr}=\frac{v}{3} \lambda _0 \bigg (\frac{R}{R_0} \bigg )^{1/3},
\end{eqnarray*}

where *v* is the particle speed, *R* is the particle rigidity and $\lambda _0$ is a reference mean free path at $R_0\equiv 1$ GV. Here $\lambda _0$ indicates the ability of the shock to confine particles: smaller values of $\lambda _0$ lead to better confinement and a more efficient acceleration of particles. The rigidity dependence ($R^{1/3}$) follows the derivation by Jokipii *et al.* [24]. However, we acknowledge that the spectral index of amplified turbulence near shocks can deviate from the Kolmogorov value [38], resulting in different rigidity dependencies of the diffusion coefficient. In reality, $\lambda _{rr}$ should be different across the shock [33,39]; the effective $\kappa _{rr}$ here represents the value in the upstream region where the scattering time is much longer.

Figure 3b shows the acceleration time matrix for the 9 November 2023 event based on the above theory (using the best fit $\lambda _0$ derived in panel (c)). The result is consistent with that shown in Fig. 3a. As indicated by the colour gradient, the required acceleration time does not vary significantly with initial energy, but increases substantially with final energy. That is, protons with higher energies need more time to be accelerated.

Figure 3c shows the acceleration time, taking into account the actual event spectra. Specifically, we consider seed particles without a single energy, but rather with a distribution similar to the VD particle spectrum (blue dots in Fig. 2c), which are accelerated to have the final spectrum of the IVD particles (orange dots). Here we have assumed that the spectra of the first-arriving particles may represent the spectra close to the acceleration site, as they experienced the least scattering. In fact, the seed particle energy is not important for the results, while the final energy is more critical (Fig. 3b). Moreover, transport effects such as adiabatic cooling could modify the particle spectra. But, the effect is only significant for low-energy particles below about 10 MeV [28], while the IVD particle spectrum is mostly above this energy range. Meanwhile, the acceleration time also depends on $\lambda _0$, as shown by the range of the coloured band. Generally, a smaller mean free path results in a shorter acceleration time for a given final energy, implying that shocks that better confine particles can better accelerate them.

Type-II radio bursts are signals of shock particle acceleration; therefore, the possible initial time of DSA acceleration ($\tau _0$) can be approximated as the start of the type-II radio emission [40]. However, for the events on 24 December 2023 and 31 December 2023, the only possible CME associated with the SEP event occurred shortly before the flare that was closest to the $t_0$ derived from the VDA. A possible scenario to explain this ‘confusing’ situation is that the flare-accelerated particles served as seed particles that were further accelerated by the shock that erupted earlier (e.g. [41]). Thus, it is also plausible to assume the flare peak time as $\tau _0$. We therefore consider both possibilities (type-II start, flare peak) as $\tau _0$ and deduce the required acceleration time of the SolO-observed IVD particles as $\tau _a(E^{\prime }) = t_{\text{release}}(E^{^{\prime }})-\tau _0$, where $t_{\text{release}}(E^{^{\prime }})$ is the derived release time (Fig. 2b). Results of $\tau _a(E^{\prime })$ derived from two possible choices of $\tau _0$ are plotted as error bars in Fig. 3c and the data can be fitted with a fixed $\lambda _0$ (listed in Table 1), shown by the dashed lines. The theoretical release time ($\tau _0$ plus $\tau _a$ fitted from $\lambda _0$) for the first event is also plotted in Fig. 2b as the black dotted curve, which agrees nicely with the observation-derived release time (orange circles).

All three events show consistent values of $\lambda _{0}$ around 10$^{-4}$ au. Although more statistics are needed for a more general conclusion, this indicates common characteristics of the DSA process, e.g. $\lambda _0$ is determined by the excitation of Alfvén waves near the shock front by streaming protons [38]. Consequently, $\lambda _0$ near the shock front derived here is much smaller than that under quiet solar wind conditions (0.4 au derived by Li *et al.* [38]). We further derive the diffusion coefficient $\kappa$ from $\lambda _0$ to be $\sim\! 10^{13}$ m$^2$/s between 1 and 100 MeV. The values are generally a few times higher than those derived by Li *et al.* at 0.18 au for 1–10 MeV protons under shock conditions, while the results are more comparable at 100 MeV. Further investigations are needed for understanding the difference in $\kappa$ derived from the two independent methods.

For the 9 November 2023 and 24 December 2023 events, we can compare the derived $\lambda _{0, {\rm release}}$ ($< {\sim }0.3$ au; see Table 1) with the *in situ* mean free path obtained at the shock when it arrived at SolO. Following the e-folding time method derived from ESP flux [42], we obtain the local parallel mean free path $\lambda _{\parallel , {\rm SolO}}$ in the upstream shock region at SolO. The results are shown as solid lines in Fig. 3d where $\lambda _{\parallel , {\rm SolO}}$ is in the range 0.003–0.02 au for the two events and varies slightly with energy. The radial mean free path can be written as $\lambda _r=\lambda _\parallel \cos ^2 \Psi$, where $\Psi$ is the angle between the local magnetic field direction and the radial direction. Based on the IMF observations, we calculate $\cos ^2 \Psi$ as approximately 0.5. Wang *et al.* [43] considered the radial dependence of the particle’s diffusion coefficient as $\kappa \propto D^\beta$ with *D* the distance to the Sun and $0\le \beta <2$. Consequently, $\lambda _{r, {\rm release}}$ near the Sun (at the release site) can be deduced from the local $\lambda _{\parallel , {\rm SolO}}$ at the location of SolO shown as dashed lines in Fig. 3d. Compared to $\lambda _r$ derived from the acceleration time (dots), $\lambda _{r, {\rm release}}$ can be aligned to the same magnitude (although at lower energies) with $\beta \sim 1.6$ and $\beta \sim 0.8$ for the 9 November 2023 and 24 December 2023 events, respectively (see Table 1). These results are roughly in agreement with Chen *et al.* [44], who obtained the radial gradient of the parallel diffusion coefficient $\kappa _\parallel \propto D^{1.17}$ using the measured magnetic turbulence power spectra in the inner heliosphere. The difference between our results and theirs can be attributed to the difference between the quiet IMF condition and the turbulent conditions at the shock front. Note that calculation of *in situ*  $\lambda _\parallel$ using ESP observations for the 31 December 2023 event is not possible because another SEP event occurred shortly after the onset of this event.

It is worth mentioning that the DSA mechanism could also be applied to gradual plasma compressions without a true shock discontinuity [45]. Observational evidence has revealed energetic particle events potentially associated with such compressed regions, even in the absence of strong CMEs or shocks [46]. If the development of shock formation is slow, compression wave acceleration can also contribute to the observed IVD feature in these events.

### Connectivity change during the shock propagation

**Figure 4.**
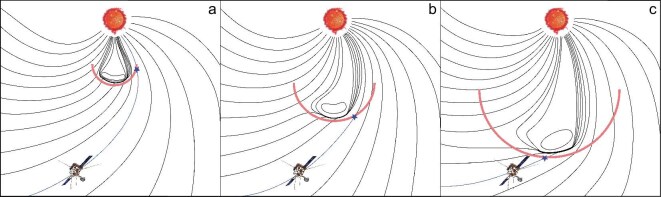
Sketch depicting the connectivity scenario that could potentially explain the later incremental arrival of higher-energy protons to the observer. The blue star marks the cobpoint, which is a point at the shock (marked by the red curves) that is magnetically connected to the observer. See the text for further details.

We also investigate the alternative explanation on how the evolving magnetic connection between the observer and shock front may cause the observed IVD. Under nominal solar wind conditions, the point at the shock that is magnetically connected to the observer, defined as the cobpoint [47], moves along the front of the shock eastward during its propagation. During this process, the observer is connected to different regions of the shock that may have different acceleration efficiencies [48]. Figure 4 shows a sketch of the simplified scenario for the 9 November 2023 event, depicting the evolution of the shock (red curve) driven by the CME (simplistically represented as a bubble). The cobpoint (blue star) slides along the shock front over time with changing magnetic connection to the observer (SolO), depicted by a blue curve in each panel.

The acceleration efficiency of the shock is generally believed to be higher close to the shock nose, while it decreases towards the shock flank, although detailed modelling of the shock evolution shows that the shock parameters (Mach number, compression ratio, etc.) may change significantly both spatially and temporally as the CME propagates through the heliosphere [49]. Assuming the simple case that the early-connected shock flank is less efficient in the SEP acceleration while the later-connected shock nose is a more efficient accelerator, Fig. 4 provides a possible explanation for the observed IVD feature in the 9 November 2023 event where the CME width and direction are derived based on GCS fitting.

Considering the connectivity changing from the flank to the nose, we check the relative angular information of all events. Table 2 shows the separation angle between SolO and the solar source (i.e. the flare associated with the SEP) and the calculated angle between SolO’s magnetic footpoint and the source. For both angles, we observe that IVD features are not restricted to a specific range of longitudinal separations, thus implying that the connectivity model may not serve as a general explanation for all events. Besides, for events with a short duration of IVD and a small radial distance (${<} 0.2$ au) upon the derived release time, the role of connectivity change should not be significant.

Furthermore, to quantify the observed IVD features, we would need a combined approach to model the shock evolution in both time and space as well as particle acceleration and transport processes [50]. Kouloumvakos *et al.* [15] made such an attempt to model the event the PSP observed on 5 September 2022 at about 15 solar radii, which was reported first by Cohen *et al.* [14], who suggested that the later released higher-energy particles do not have enough time to overtake the earlier released lower-energy ions before reaching the spacecraft that was sufficiently close to the Sun. Kouloumvakos *et al.* [15] modelled both the shock and particles simultaneously and further proposed that the PSP was initially connected to the weaker part of the shock and later to a strengthened shock with higher acceleration efficiency. The connectivity change resulted in the observed later arrival of particles with higher energies at the PSP. But, the modelled particle intensity was higher than observed and the particle profile detected at Solar Orbiter was not well reproduced. A more consistent Sun-heliosphere-shock-particle modelling effort would be necessary to better reveal the general nature of IVD particles, such as that by Ding *et al.* [51], who successfully reproduced the observed IVD signatures by SolO on 7 June 2022.

Additionally, as we noted for the 9 November 2023 event, two CMEs were launched within a short time window. Interacting CMEs may result in possible changes in the shock angle [52] and thus impact the evolution of the connectivity, which becomes a much more complex scenario. In fact, we cannot rule out the presence of a shock driven by the northern CME, which may have interacted with the main southern shock, giving rise to a more complex particle acceleration process due to shock interactions [53]. Such modelling effort would require many input parameters that are currently not easy to validate against observations and is thus not pursued further in this study.

## CONCLUSION

In summary, this study shows a new type of solar energetic proton event observed by SolO that has an unusual inverse velocity dispersion structure (at energies above a few mega-electron-volts) during the event onset in addition to the typical velocity dispersion at lower energies. This phenomenon can be explained to be caused by the delayed release of particles with increasing energies. We analysed the travel path and release time of particles for both VD and IVD components and quantified the IVD release time as a function of proton energies. We found that the VD proton release time is consistent with the flare X-ray bursts and initial type-II radio emission, which indicates that these particles are likely associated with the flare process or early-shock formation close to the Sun. For IVD protons, the release time is much later when the shock is at a solar distance between about 0.05 and 0.2 au and it increases with particle energy.

These observations can be explained by two different mechanisms (or a combination of them). First, it takes a longer time for higher-energy particles to be accelerated, as required by the diffusive shock acceleration mechanism, based on which we derived the shock parameters during the acceleration process. Second, as the shock propagates outward, the observer’s magnetic connection to the shock front changes. In the case of an IVD event, the observer gets connected to regions with increasing acceleration efficiencies. However, for the events studied here, connectivity theory may not be a general explanation as it requires restricted separation angles between the observer and the source.

To conclude, this new trait in the high-energy range of solar proton events helps us to innovatively retrieve physical parameters during the acceleration process directly from observations, in particular the actual acceleration time scales that cannot be observed directly. Nevertheless, more coordinated observation and modelling efforts are needed to constrain the two different mechanisms, their interlink and contributions to the observations. In particular, shock-related acceleration can be modelled with magnetohydrodynamics (MHD) shock modelling coupled with particle transport simulations [15,51]. Such models can provide key parameters, including evolution of shock properties, magnetic connectivity and energy-dependent release profiles, which can then be directly compared to the *in situ* particle and plasma observations. We emphasize that the particle radiation environment during solar eruptions is dynamically varying in both spatial and temporal dimensions and that radiation assessment should be tailored for different observers, taking into account the relative position of the observer to the acceleration source and the evolution of the source. Last but not least, IVD features have not been reported prior to recent observations because they may have been overlooked, so the development of the new instrumentation with better time and energy resolution has opened new opportunities to discover new phenomena in the observations.

## METHODS

### Velocity dispersion analysis

Under the assumption that at the beginning of the event the first-arriving particles are released simultaneously and travel along the same path length, we expect that particles with higher energies would arrive earlier. This has been frequently observed before and is called velocity dispersion [7,23,54,55]. The VDA often assumes a small cross-field scattering process that is supported by the high anisotropy of the SEPs upon the onset of this event, as shown in the online supplementary material. To determine the release time and path length of particles with the velocity dispersion feature, the following function is often applied:


(7) \begin{eqnarray*}
t_{\text{onset}}(E)=t_{\text{0}}+8.33\frac{\text{min}}{\text{au}}\, \frac{L_0}{\beta (E)}.
\end{eqnarray*}

Here $t_{\text{onset}}(E)$ (in units of minutes) is the observed SEP onset time (as explained later) for particles with kinetic energy *E*, $\beta (E)=v(E)/c$ is related to the velocity of the particles, $t_{\text{0}}$ (in units of minutes) and $L_0$ (in units of 1 au) are the initial release time and the travel path to be derived from this function. Based on $\beta (E)$ (*x* axis) and the observed $t_{\text{onset}}$ (*y* axis), we can linearly fit the above function, using the orthogonal distance regression method. The uncertainty in energy arises from the energy bin width of the EPD, while the uncertainty in the time originates from 5-min intervals of the particle flux data. This method allows us to determine $L_0$ as the slope and $t_0$ as the intercept with the *y* axis. It should be noted that the EPD sunward telescope is not strictly field aligned due to the changing direction of the IMF so that the pitch angle of observed particles varies over time. Consequently, the onset particles at different energies may correspond to different pitch angles. These factors may introduce some uncertainties in the derived $L_0$ and $t_0$.

In the above process, the cumulative sum (CUSUM) quality-control scheme [56] was applied to determine the onset time $t_{\text{onset}}$ for particle fluxes at each energy of the VD part.

Based on the determined $t_0$, we identify the flare and CME that occurred nearest (normally within 1 h) as the most likely responsible cause of the SEP event (see also the online supplementary material for the flare and CME observations).

### Inverse velocity dispersion analysis

For particles with IVD features, we assume that their release time $t_{\text{release}}(E^{^{\prime }})$ and path length $L(E^{^{\prime }})$ change as a function of energy (expressed as $E^{^{\prime }}$, to be different from *E* used for the VDA). The particle release time $t_{\text{release}}(E^{^{\prime }})$, onset time $t_{\text{onset}}(E^{^{\prime }})$, velocity $\beta (E^{^{\prime }})$ and propagation distance $L(E^{^{\prime }})$ still follow the expression


(8) \begin{eqnarray*}
t_{\text{onset}}(E^{^{\prime }})=t_{\text{release}}(E^{^{\prime }})+8.33\frac{\text{min}}{\text{au}} \, \frac{L(E^{^{\prime }})}{\beta (E^{^{\prime }})};
\end{eqnarray*}

however, for IVD particles, the low signal-to-noise ratio and inadequate data during the pre-event background period often result in poor performance and unreliable results of the Poisson-CUSUM algorithm that was used for the VDA. Here we opt to manually determine the onset time based on the two-dimensional histogram of the energy-dependent particle flux. To minimize uncertainties, this process was repeated multiple times until the result stabilised.

With both $t_{\text{release}}(E^{^{\prime }})$ and $L(E^{^{\prime }})$ being variables, it is not feasible to directly fit the above function. So we use an iteration process to solve the above function. We assume that the initial value of $L(E^{^{\prime }})$ equals $L_0$ derived from Equation (7) and calculate $L(E^{^{\prime }})$ as the CME shock propagates away from the Sun.

First, based on the initial properties of the CME derived from remote-sensing observations, we use the drag-based model (DBM; [57]) to propagate the shock from 17 solar radii into interplanetary space. The DBM is a semi-empirical tool for CME propagation, assuming that beyond $\sim$15 solar radii, the dynamics is governed solely by the interaction between the CME and the ambient solar wind. Despite its simplicity, the model has proven to perform equally well compared to other more complex MHD models [58,59]. Detailed input CME and solar wind parameters for the model are given in the online supplementary material.

Second, with the consideration that the particle release site (i.e. the shock front) evolves with time (as does energy $E^{^{\prime }}$) when the shock moves outward, we then derive the shock distance at different release times $t_{{\rm release}}(E^{^{\prime }})$ with the path length $L(E^{^{\prime }})$ modified at each $t_{{\rm release}}(E^{^{\prime }})$ using the following iteration process.

1. In the first step, we assume that $L(E^{^{\prime }})$ is approximately $L_0$ as derived from Equation (7). Then Equation (8) can be solved to obtain $t_{{\rm release}}(E^{^{\prime }})$ for $E^{^{\prime }}$.
2. At the above derived time $t_{{\rm release}}(E^{^{\prime }})$, we obtain the shock propagation distance from the Sun $R(t)$ using the DBM. We then modify the particle propagation path as $L(E^{^{\prime }})-R(t_{{\rm release}}(E^{^{\prime }}))$ to account for the shortened distance of the particle propagation path compared to that of the initial release (see below for further explanations).
3. With the modified path, we re-calculate $t_{{\rm release}}(E^{^{\prime }})$ from Equation (8), which corresponds to another $R(t)$ that can be different from step 2 that will further change the propagation path.
4. Steps 2–3 are repeated until $t_{{\rm release}}(E^{^{\prime }})$ and $L(E^{^{\prime }})$ converge (i.e. newly derived values equal to those derived in the previous step).
5. The uncertainty of $t_{\text{release}}(E^{^{\prime }})$ is calculated using the error propagation method, accounting for the observational time interval and energy range.

In the above approach, we assumed that the propagation distance of the shock corresponds to the shortened distance of the SEP path length because, for the derived release time $t_{{\rm release}}(E^{^{\prime }})$, normally, the CME was still close to the Sun (mostly within 0.3 au) where the interplanetary magnetic field has a much higher radial component.

## Supplementary Material

nwaf348_Supplemental_File

## Data Availability

All data used in this study are available on NASA’s website (https://cdaweb.gsfc.nasa.gov/).
