# Supplementary material for: The delayed arrival of faster solar energetic particles as a probe into the shock acceleration process
Source: Natl Sci Rev. 2025 Aug 26;12(10):nwaf348. doi: 10.1093/nsr/nwaf348 (PMC12485618; doi:10.1093/nsr/nwaf348)
Supplement: nwaf348_Supplemental_File [file nwaf348_supplemental_file.pdf]

National Science Review
: output,
doi: https://doi.org/

The delayed arrival of faster solar energetic particles as a probe into diffusive shock acceleration process: Supplementary Information

Dataset

This study is based on data from several space missions observing from different longitude and radial distance from the Sun. We used in-situ and remote-sensing data from Solar Orbiter (SolO), as well as measurements from the Solar-Terrestrial Relations Observatory Ahead (STEREO A, 1) and near-Earth observatories like the Geostationary Operational Environmental Satellite (GOES, 2), and the Solar and Heliospheric Observatory (SOHO, 3).

The unusual solar proton events are measured by the Energetic Particle Detector (EPD, 4; 5) instrument suite onboard SolO, which provides comprehensive ion observations across a broad energy range. In this work, observations of vital energy ranges of IVD events are from the Electron Proton Telescope (EPT) and the High-Energy Telescope (HET) of EPD. EPT measures ions from 48 to 6100 keV while providing anisotropy information from four different viewing directions, which point sunward, anti-sunward, north and south. Generally for scatter-free and scatter-poor events, first particles arrive primarily from the direction of the Sun so that the sunward-looking telescope measures the earliest onset of particles (see Supplementary Information). Therefore, this study mainly uses the sunward-looking data for the onset analysis.

Meanwhile, HET can discriminate between different elements and measures ions from 6.8 MeV nuc^{-1} to more than 100 MeV nuc^{-1}, with the upper energy limit depending on the ion species. HET also provides four fields of view that share pointing directions with EPT.

SEPs are tightly associated with the eruption of solar flares which are often accompanied by CMEs and shocks (6). Observations of solar flares are carried out by the Atmospheric Imaging Assembly (AIA, 7) onboard Solar Dynamic Observatory (SDO, 8) in multiple EUV wavelengths and cross-checked for the higher energy ranges with the Spectrometer/Telescope for Imaging X-rays (STIX, 9) onboard SolO when possible. The SDO/AIA flare observations of the 2023-11-09 event are given in the Section: Flare HXR and radio observations and Figure 1 of the main text.

Meanwhile, we characterise the associated CMEs based on coronagraph images taken by the C2 and C3 coronagraphs of the Large Angle and Spectrometric COronagraph (LASCO, 10) on board the SOHO spacecraft and COR1 and COR2 in the Sun Earth Connection Coronal and Heliospheric Investigation (SECCHI, 11) instrument suite provided by the STEREO-A spacecraft. The SOHO LASCO CME Catalogue[1] has been employed to help identify the flare and CME associated with the SEP event considering the closest eruption time to the SEP release time (explained in Method section of the main text). For the 2023-11-09, 2023-12-24 and 2023-12-31 SEP events, their associated flare and CME information is given in Table 1 of the main text. Coronagraph images from both SOHO and STEREO-A are combined to reconstruct CMEs using the GCS modelling (12) approach to obtain the initial height, timing, speed and direction of the CME (at about $\sim$ 10 solar radii). The exact parameters of the fitted CMEs are given in the Supplementary Information.

Additionally, radio observations, serving as important indicators of shock acceleration and particle propagation, are provided by the Radio and Plasma Wave Science (WAVES, 13) measurements onboard WIND spacecraft, and the Radio and Plasma Waves (RPW, 14) data onboard SolO are taken as a reference to better identify the timing of the events. The WIND

[1] https://cdaw.gsfc.nasa.gov/CME_list/

Received: ; Revised: ;
Accepted:

© The Author(s) . Published by Oxford University Press on behalf of China Science Publishing & Media Ltd. This is an Open Access article distributed under the terms of the Creative Commons Attribution License (http://creativecommons.org/licenses/by/4.0/), which permits unrestricted reuse, distribution, and reproduction in any medium, provided the original work is properly cited.

observations (which are clearer than RPW plots) are given in the Supplementary Information. The time durations of the Type II radio emission for the 2023-11-09, 2023-12-24 and 2023-12-31 SEP events are given in Table 1 of the main text.

Other instruments onboard SolO are used to understand the interplanetary plasma and magnetic environment where particles propagate through (see Figure 1(c) of the main text). Solar wind parameters such as bulk speed, proton density and proton temperature are provided by the Solar Orbiter Solar Wind Analyser (SWA, 15) suite, while the interplanetary magnetic field is measured by the Solar Orbiter magnetometer (MAG, 16).

Flare HXR and radio observations

The flare closest to the VD release time (11:39 ± 7 mins) of **the** 2023-11-09 event was a C2.6 class flare, lasting from 10:45 UT to 11:29 UT, peaking at 11:05 UT **(time has been subtracted considering the light travel time).** Fig. 1(b) shows the hard and soft X-ray count rates as recorded by SolO/STIX, and the time axis is shifted to 1 AU to campare with the solar radio burst observed at 1 AU. The flare was located at W04, S10 based on SDO/AIA observations. The longitudinal separation between the flare and Solar Orbiter's magnetic footpoint as derived from the observed solar wind speed of 350 km/s was 21.3 degrees. The longitudinal separation between the flare and the Earth's magnetic footpoint as derived from the observed solar wind speed of 680 km/s was 33.3 degrees.

Although the CME speed and flare classes are not among the highest on record, strong solar radio bursts were observed. In the observed frequency bands from WIND/WAVES, two slow-drifting Type II radio bursts and a fast-drifting Type III radio burst at higher frequencies are presented in Fig. 1(a). The two Type II radio bursts correspond to two closely occurring eruptive C-class flares and their related CME shocks. The later one started at 11:04 UT spanning ~1 MHz to 20 kHz. Together with the earlier occurring Type III radio burst, this strongly indicates the particle acceleration processes from the flare-CME event chain.

Table 1 in the main text shows the timing of flare X-rays and radio bursts for the 2023-11-09, 2023-12-24 and 2023-12-31 events.

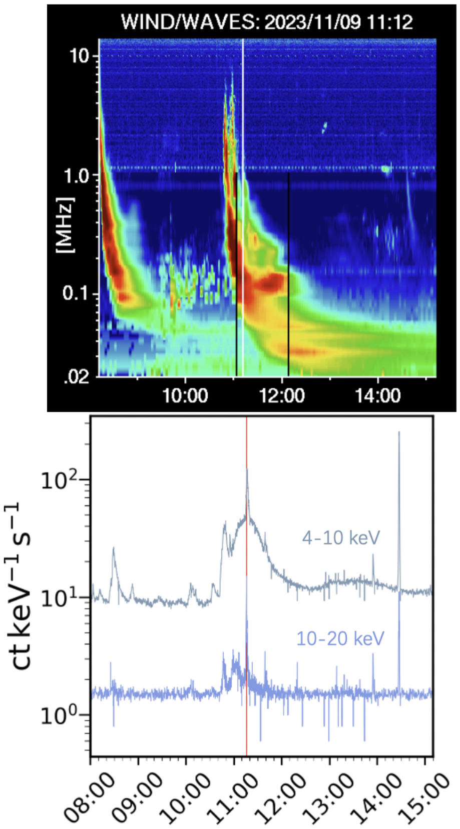

Figure 1: (a) Type II and Type III radio burst associated with the 2023-11-09 event, observed by WIND/WAVES, with the white line indicating the Type II start time corresponding to the later flare (S10W04). (b) Soft X-ray (4-10 keV) and hard X-ray (10-20 keV) observations from SolO/STIX across two channels, with red line marking the peak of each channel. For consistency, the time axis shown in the panels represent the light arrival time at 1 AU, whereas the times mentioned in the text have been adjusted by subtracting 8.33 minutes.

CME propagation derived from DBM

In this study, the drag-based model (DBM; 17) is employed to determine the kinematics of CME

shocks, chosen for its high efficiency. The analytical solutions for acceleration, velocity and height are given by

$$a(t) = -\gamma(v(t) - v_{sw})|v(t) - v_{sw}|, \quad (1)$$

$$v(t) = \frac{v_0 - v_{sw}}{1 + \gamma(v_0 - v_{sw})t} + v_{sw}, \quad (2)$$

$$r(t) = \frac{1}{\gamma} \ln\left[1 + \gamma(v_0 - v_{sw})t\right] + v_{sw}t + r_0, \quad (3)$$

where r_0 is the initial radial distance, v_0 is the initial CME speed at r_0 in km/s, v_{sw} is the ambient solar wind speed in km/s, and γ is the drag parameter that determines the rate at which the CME **interacts with** the solar wind. The input parameters for the four events analysed in detail are presented in Table 1.

Inputs related to shock geometry such as r_0 and initial time, are derived from the Graduated Cylindrical Shell (GCS) modelling (see 12). To re-construct the 3D geometry, propagation direction and kinematics, GCS requires white-light observations of a CME from at least two viewpoints. The software developed by Forstner (19) used in this study, adapts combinations of COR2-A on STEREO-A and LASCO C2/C3 on SoHO. For shock simulation, the fitting can be accomplished by setting the half-angle to zero and the aspect ratio close to one (20).

Although DBM is used to describe the kinematic evolution of CME magnetic structures, Vršnak et al. (18) found a parameter range of γ values for which the arrival times of shocks aligned well with the results from ENLIL shock front tracking (see also 21). Studies from e.g., Dumbovic et al.2018, Temmer et al.2015, Guo et al. 2018 (22; 20; 23), have all applied the DBM in CME shock simulations with different empirical γ values in different situations. Here in our work, we adapt $\gamma = 0.1 \cdot 10^{-7}\text{km}^{-1}$ which is consistent with the values used to compare DBM and ENLIL shock simulation in Vršnak et al. 2014 (18).

Using GCS fittings on the early stage shock, the shock speed v_0 can be determined from quasi-GCS measurements (20). Figure 2(a) shows examples of shock fitting for the 2023-11-09 event at two time points, derived from running-difference white-light images. These fittings are utilized to estimate the shock speed at the respective times.

Due to the difficulty of measuring ambient solar wind speed v_{sw} throughout the CME propagation, various methods have been employed to determine the value of v_{sw} when using DBM. In our study, for IVD candidates linked to a CME that has passed SolO, we directly use solar wind speed measurements of the shock **upstream** from SWA measurements. For those not impacted by the CME shock at SolO, we use inferred values from the DBEMv4 solar wind module[2] which have significant uncertainties. So both the modelled and measured solar wind speeds have been assigned a 20% uncertainty, as shown in Table 1.

By applying the DBM, the energy-dependent release phase of IVD particles in 2023-11-09 event within the CME propagation can be obtained, as shown in Fig. 3. The IVD particles, ranging from 11 to 53 MeV, were released over a duration lasting more than 4 hours. These particles are released sequentially from lowest to highest energy, when the shock was below 0.16 AU. This feature of sequential release according to energy is evidence of the diffusive acceleration mentioned in the main text, as well as an explanation for the IVD phenomenon.

Directionality of SEPs during the 2023-11-09 event

Particle directionality at the onset of the SEP events is shown in Fig. 4 with the 2023-11-09 event as an example based on measurements from the four apertures of the EPT and HET. The top four panels display the intensity colour coded multiplied by E^2 for the combined EPT and HET measurements of the four fields of view (i.e., Sun, anti-Sun, north, and south). The pitch angle at the centre of each aperture is indicated by a dash-dotted curve within each panel, scaled to the right-hand y-axis. A faint dash-dotted line marks 90 degrees as a reference. The earliest and most distinct IVD features were noted in the sunward-facing EPT and HET at approximately 15:00 UT on 9 November, with similar observations following about an hour later in the south-facing telescope. In contrast, such phenomena in the anti-sunward and north orientations were only detected in the HET energy channels.

First-order anisotropy A is defined as the following equation:

$$A = \frac{3\Sigma_i I(\mu_i) \cdot \mu_i \cdot \delta\mu_i}{\Sigma_i I(\mu_i) \cdot \delta\mu_i} \quad (4)$$

where $I(\mu)$ is the pitch-angle-dependent intensity measured by the i viewing direction and μ is the average pitch angle cosine of the direction (24). The coverage of the pitch angle depends on the orientation of the magnetic field

[2] https://swe.ssa.esa.int/graz-dbem-federated

**No.**	**Date**	**Initial time**	r_0 (R_{Sun})	ϕ ()	θ ()	v_0 **(km/s)**	$v_{\rm sw}$ **(km/s)**	γ ($\cdot 10^{-7}$ **km**$^{-1}$)
**1**	2023-11-09	14:57	17±1.7	6	17	870±87	350±70	0.1∓0.05
**2**	2023-12-24	16:54	17±1.7	340	79	740±74	300±60	0.1∓0.05
**3**	2023-12-31	13:54	17±1.7	101	36	830±83	400±80	0.1∓0.05

Table 1: DBM input parameters for the 3 events analysed in detail. γ values between 0.1–0.2×10^{-7}km^{-1} are found to match best for simulating the CME shock-sheath structure (see 18). In this study, the uncertainties for r_0,v_0 are 10%, for $v_{\rm sw}$ 20% and for γ 50%.

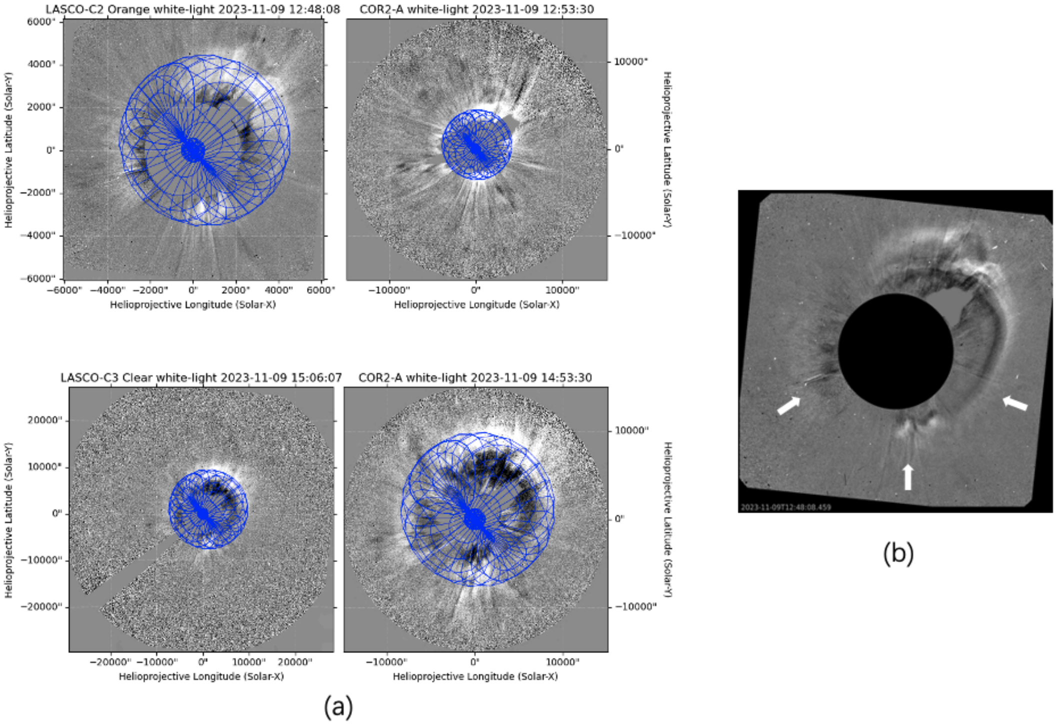

Figure 2: (a) GCS fitting for the shock (blue mesh) using simultaneous running difference white-light image from COR2-A/STEREO-A and LASCO/SoHO at ~12:50 (first row) and ~15:00 (second row) on 2023-11-09. (b) Running difference white-light image from LASCO C2/SoHO at 12:48. Note that another flare, occurring slightly before the SEP-related flare in the northern hemisphere, is associated with a northward CME. This CME interacted with a subsequent southward halo CME, that is related to the SEP event and marked by three arrows at the image's edge.

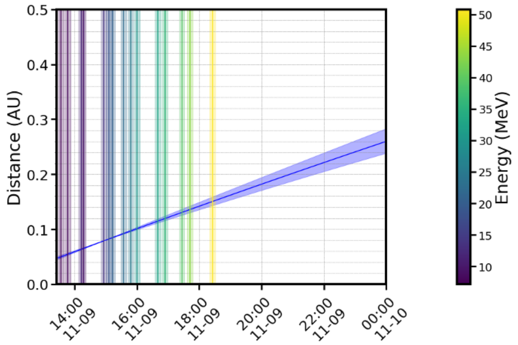

Figure 3: The derived release time and distance for IVD particles and the propagation time and distance of the DBM-modelled shock for the 2023-11-09 event. The blue line indicates distance, while the blue shadow band represents the output uncertainty due to the input uncertainties mentioned earlier (listed in Table 1). Vertical lines marked the release time $t_{\text{release}}(E^{'})$ of IVD particles at different energies, indicated by the colour bar on the right. The uncertainty of $t_{\text{release}}(E^{'})$ is calculated using error propagation methods accounting for the observational time interval and energy range.

relative to the aperture of the telescopes. EPT and HET apertures do not cover the entire sky area and hence do not observe the complete μ-space. Consequently, the reconstruction of the pitch-angle distribution is limited by the four directions of the observations.

The last two panels present strong anisotropy of the ~1 MeV proton at the onset stage around 15:00 UT, November 9. Panel (f) shows the intensity-time profile for the 1 MeV protons from different EPT telescopes. The sunward telescope observed not only the earliest onset but also the highest flux. In panel (e), A in the onset period shows a large absolute value ($|A| \approx 2$) and lasts for approximately 6 hours. Coupled with the earliest onset of sunward particles shown in panel (f), this suggests a significant anisotropy in the arrival times of particles from different orientations. The anisotropy is negative, meanwhile the B_r shown in (see Fig. 1(c) in main text) is negative. This indicates that the initial acceleration site of these particles was at the Sun. **As the event continues, the absolute value of anisotropy converges towards zero except for a short duration of fine structure from 19:00 of Nov 11 until 00:00 of Nov 12. This is known as the reservoir effect (25) during the later phase of SEP events due to strong particle scatter process in the heliosphere.**

SEP observations of 2023-12-24 and 2023-12-31 events

Figure 5 shows the measurements and VDA/IVDA results for the 2023-12-24 event. In panel (a) we depict the dynamic spectra with the proton measurements from EPT and HET. Blue circles mark the onset time for each energy bin in the lower energy range, corresponding to the regular VD pattern, and the orange dots show the onset for the energy range in the IVD part. All onsets were selected manually. In panel (b) we plot the 3-hour-integrated spectra for both energy ranges starting from the onset of each energy range, using the same colours for VD and IVD as in panel (a). This event was observed by SolO from a radial distance to the Sun of 0.94 au and under a measured solar wind speed of 460 km/s. The results of the VDA in panel (c) suggest a release time of 16:18 UT ± 17 min, and a **travelled** path length of 1.48 ± 0.38 au, comparable with the 1.04 au calculated for the correspondent Parker spiral.

Similar to the previous figure, Figure 6 presents the measurements and VDA/IVDA results for the 2023-12-31 event. This event was observed by SolO at a radial distance to the Sun of 0.95 au and under a measured solar wind speed of 393 km/s. The VDA results suggest a release time of 12:04 UT ± 6 min, and a travelled path length of 1.33 ± 0.17 au, comparable with the 1.09 au calculated for the correspondent Parker spiral.

Information of other IVD events

As a reference, Fig. 7 and Fig. 8 show the 9 IVD events mentioned in Table 2 of the main text and location of the identified flare related to each of them. The release time derived through VDA, combined with EUV images from The Extreme Ultraviolet Imager (EUI) onboard SolO and white-light observations from SOHO's C2, helps determine the related eruptions. There are a few noteworthy points:

- 2022-06-07 event: the onset of VD particles is divided into two segments, each showing a strong linear relationship, leading to different release times. The release time preceded the eruption time adapted in Table 2 by two and a half hours, and this eruption was the most intense flare within this time period.
- 2022-06-26 event: Prior to the inferred release time, there were a series of eruptive events, including two flares with close longitudes in the southern and northern hemispheres, respectively, and a filament eruption near the eastern

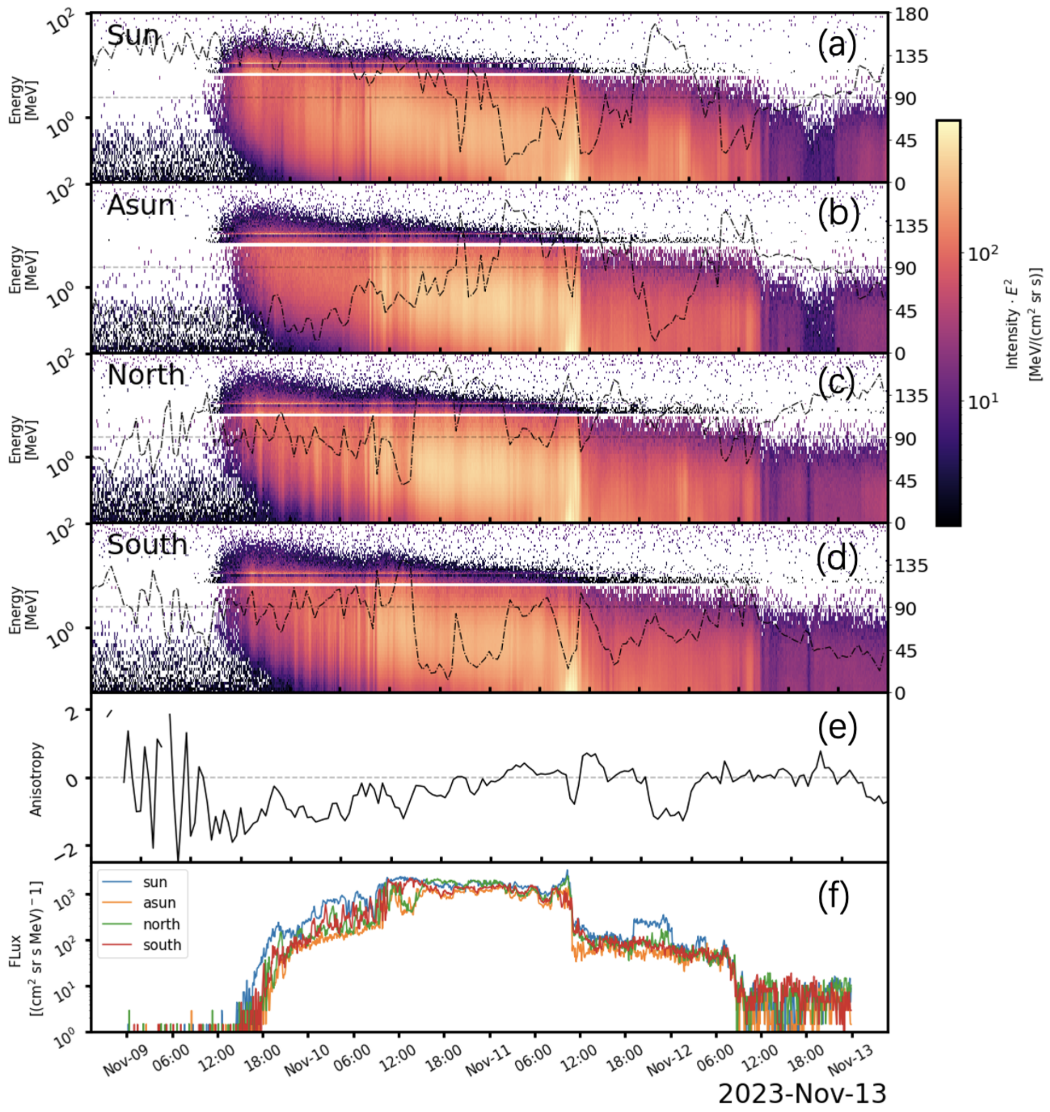

Figure 4: Top 4 panels are EPT observations from different telescopes for protons, as indicated in the upper-left corner of each panel. Pitch angle is also shown as a dot-dashed line and is represented by the scale on the right-hand axis. (e) shows anisotropy of the first arrival energy, ~1 MeV, while (f) shows the intensity-time profile of the same energy channel.

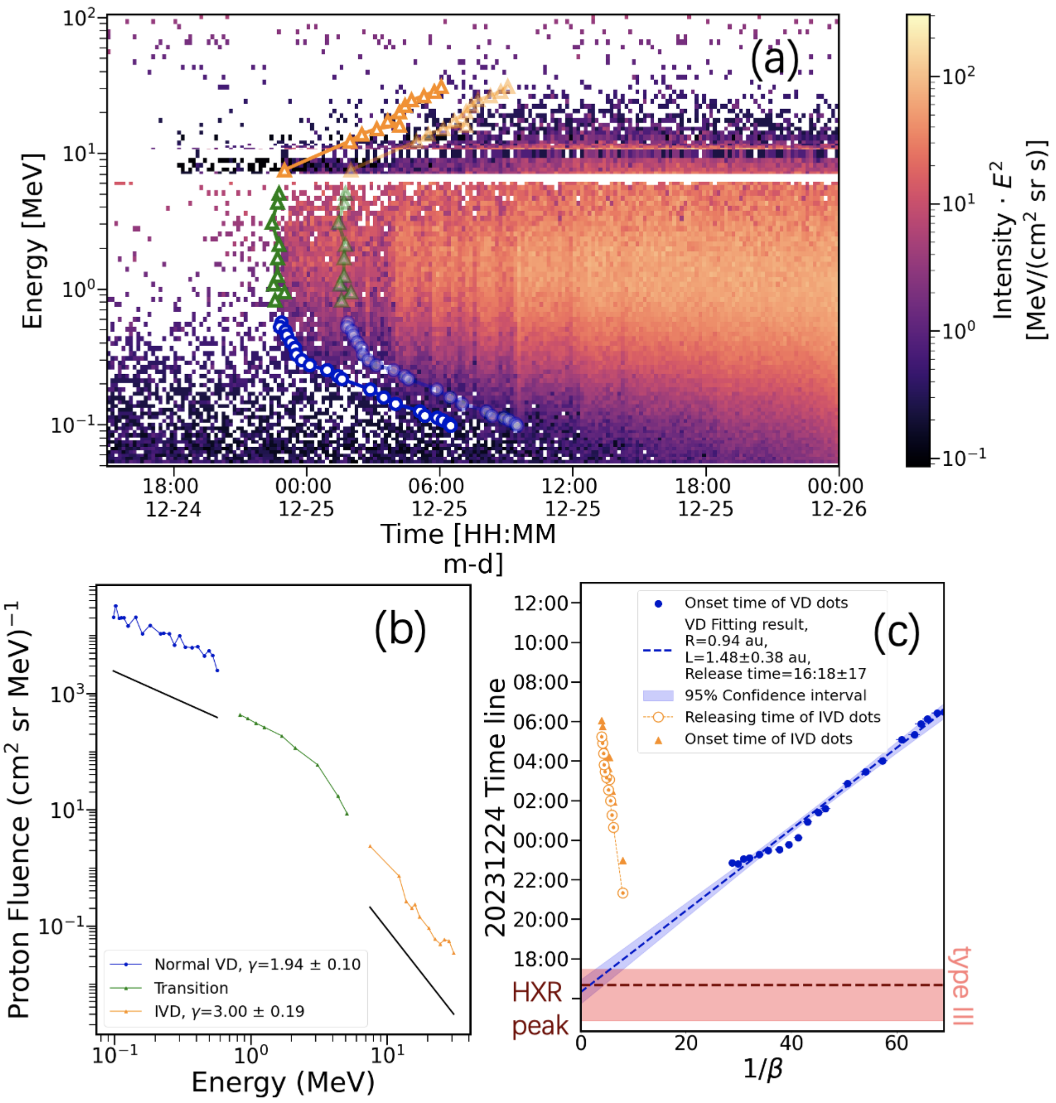

Figure 5: (a) Dynamic spectra of the early phase of the 2023-12-24 event; (b) The release time and path length result from VDA of protons under ~0.7 MeV and from IVD release time analysis of protons above ~ 7 MeV and (c) The 3-hour-integrated energy spectra of proton measurements, starting from the onset of each energy range. In (a), the onset time for VD particles is marked with blue circles, while the onset time for IVD particles is indicated with orange triangles (for onset time determination see Method in the main text). The first arrival energy within the transition range between VD and IVD energy is marked with green triangles. The onset time is used in panel (b) following VDA method (see Method in main text) to derive the release time and path length of energetic protons shown in the legend. Timing of the M2.6 flare hard X-ray (~ 10 keV) and related radio bursts shifted to the solar surface (subtracting 8 min accounting for the time of photons reaching SolO at 0.94 au) is marked by horizontal dashed lines and pink band, respectively. In (c), the power-law fitting obtained for the low- and high-energy parts is marked by the black lines and the legends.

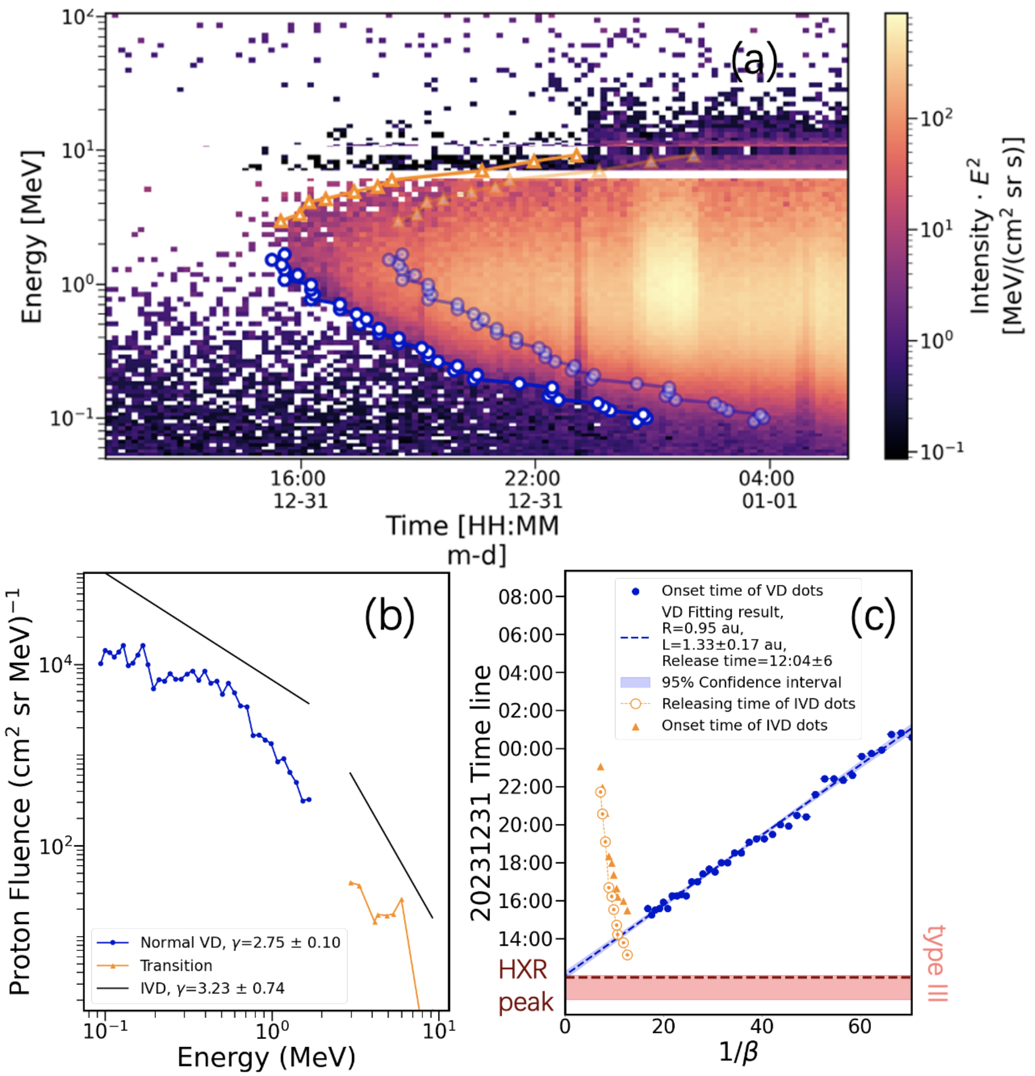

Figure 6: Same as Fig. 5 for the event on 2023-12-31.

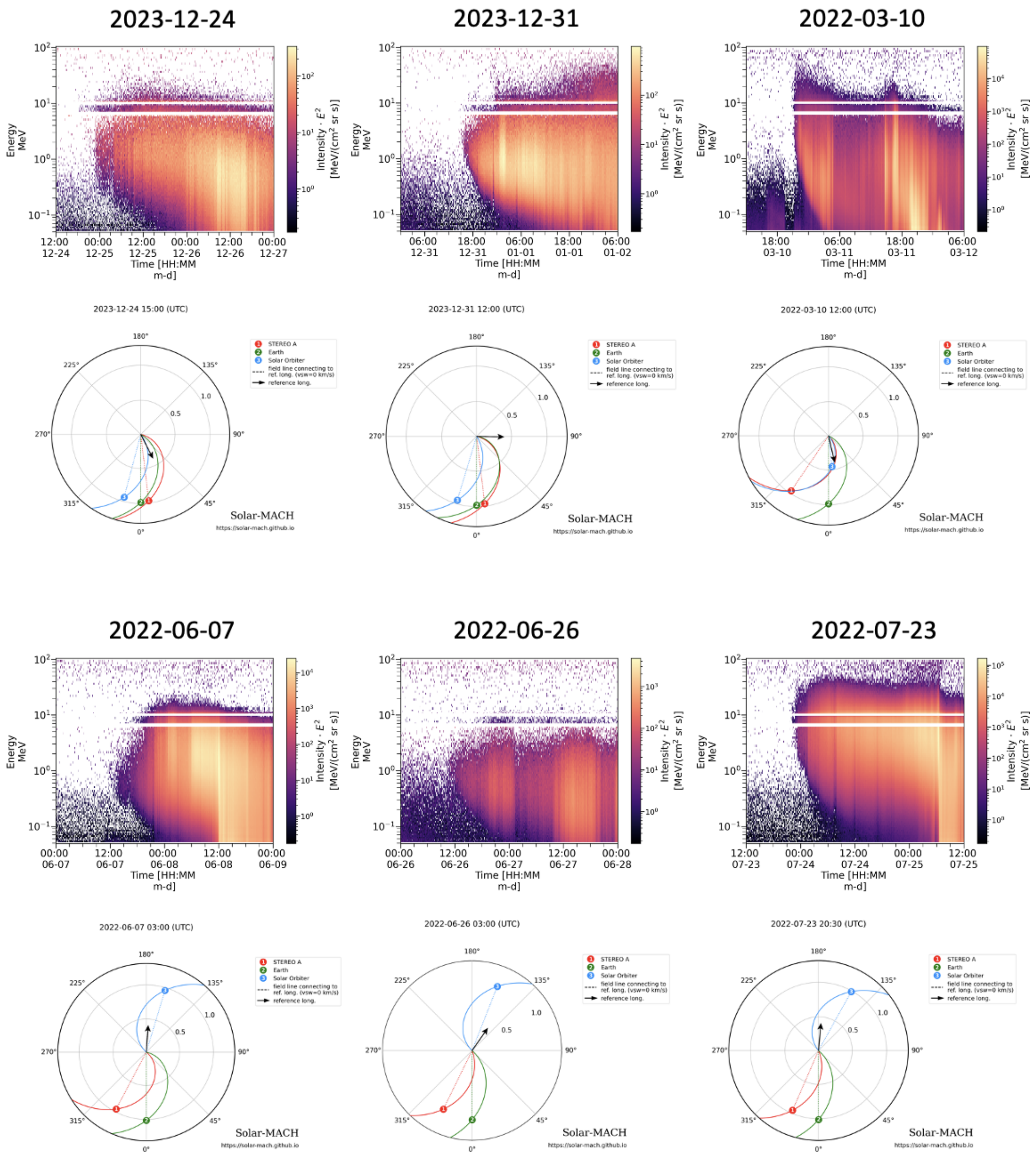

Figure 7: Dynamic spectra of 6 of the 10 IVD events listed in Table 2 of the main text, along with the configuration plot with the location of solar source. In each longitudinal configuration, the red, green, and blue markers and curves represent STEREO A, Earth, and SolO along with their respective Parker spirals. The black arrow indicates the location of the flare most likely associated with the SEP event.

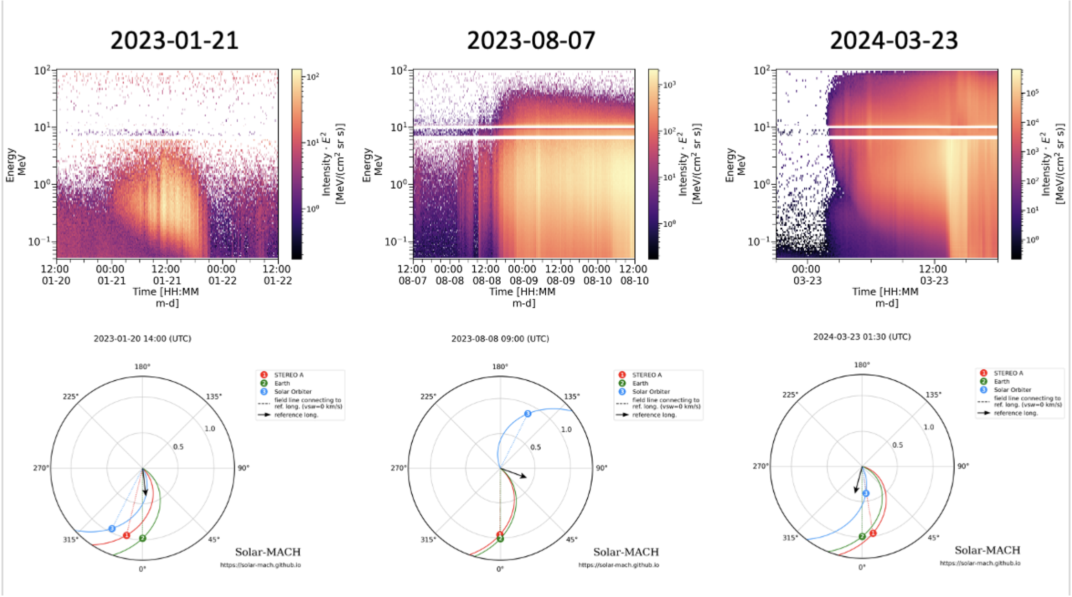

Figure 8: Same as Fig. 7 for the other 3 events listed in Table 2 of the main text, except for the 2023-11-09 event discussed in detail.

limb (from SolO's FOV). The configuration in Table 2 shows the direction of the northern flare, which was related to a CME.

- 2023-08-07 event: this event shows an unusual and complex onset phase that could not be determined using CUSUM or manual selection to identify the onset time of the VD part. Additionally, EUI observations were missing during the event, and according to X-ray observation from STIX, multiple eruptions occurred on the day of the SEP onset, plus an X-class flare the day before. The eruption directions plotted in Fig. 8 (and recorded in Table 2 of the main text) is derived from the flare location tool on the STIX quick-look website[3], which indicates the concentrated positions of eruptions.

REFERENCES

1. Kaiser ML, Kucera TA, Davila JM *et al.* The STEREO Mission: An Introduction. 2008; **136**: 5–16.
2. Space-Systems-Loral, editor. *GOES I-M Databook, Revision 1* (1996).
3. Domingo V, Fleck B and Poland AI. The SOHO Mission: an Overview. 1995; **162**: 1–37.
4. Rodríguez-Pacheco J, Wimmer-Schweingruber RF, Mason GM *et al.* The Energetic Particle Detector. Energetic particle instrument suite for the Solar Orbiter mission. 2020; **642**:A7.
5. Wimmer-Schweingruber RF, Janitzek NP, Pacheco D *et al.* First year of energetic particle measurements in the inner heliosphere with Solar Orbiter's Energetic Particle Detector. 2021; **656**:A22.
6. Reames DV. Particle acceleration at the Sun and in the heliosphere. 1999; **90**: 413–491.
7. Lemen JR, Title AM, Akin DJ *et al.* The Atmospheric Imaging Assembly (AIA) on the Solar Dynamics Observatory (SDO). *Solar Physics* 2012; **275**: 17–40.
8. Pesnell WD, Thompson BJ and Chamberlin PC. The Solar Dynamics Observatory (SDO). 2012; **275**: 3–15.
9. Krucker S, Hurford GJ, Grimm O *et al.* The Spectrometer/Telescope for Imaging X-rays (STIX). 2020; **642**:A15.
10. Brueckner GE, Howard RA, Koomen MJ *et al.* The large angle spectroscopic coronagraph (lasco). 1995; **162**: 357–402.
11. Howard RA, Moses JD, Vourlidas A *et al.* Sun earth connection coronal and heliospheric investigation secchi. *Space Science Reviews* 2008; **136**: 67.
12. Thernisien AFR, Howard RA and Vourlidas A. Modeling of Flux Rope Coronal Mass Ejections. 2006; **652**: 763–773.
13. Bougeret JL, Kaiser ML, Kellogg PJ *et al.* Waves: The Radio and Plasma Wave Investigation on the Wind Spacecraft. 1995; **71**: 231–263.
14. Maksimovic M, Bale SD, Chust T *et al.* The solar orbiter radio and plasma waves (rpw) instrument. 2020; **642**:A12.
15. Owen CJ, Bruno R, Livi S *et al.* The Solar Orbiter Solar Wind Analyser (SWA) suite. 2020; **642**:A16.
16. Horbury TS, O'Brien H, Carrasco Blazquez I *et al.* The solar orbiter magnetometer. 2020; **642**:A9.
17. Vršnak B, Žic T, Vrbanec D *et al.* Propagation of Interplanetary Coronal Mass Ejections: The Drag-Based Model. 2013; **285**: 295–315.
18. Vršnak B, Temmer M, Žic T *et al.* Heliospheric Propagation of Coronal Mass Ejections: Comparison of Numerical WSA-ENLIL+Cone Model and Analytical Drag-based Model. 2014; **213**:21.
19. Forstner JL. Gcs in python 2024.
20. Temmer M and Nitta N. Interplanetary propagation behavior of the fast coronal mass ejection on 23 july 2012. *Solar Phys.* 2015; **290**: 919–932.

[3] `https://datacenter.stix.i4ds.net/`

21. Dumbović M, Čalogović J, Martinić K *et al.* Drag-Based Model (DBM) Tools for Forecast of Coronal Mass Ejection Arrival Time and Speed. *Frontiers in Astronomy and Space Sciences* 2021; **8**:58.
22. Dumbović M, Čalogović J, Vršnak B *et al.* The Drag-based Ensemble Model (DBEM) for Coronal Mass Ejection Propagation. *The Astrophysical Journal* 2018; **854**: 180.
23. Guo J, Dumbović M, Wimmer-Schweingruber RF *et al.* Modeling the Evolution and Propagation of 10 September 2017 CMEs and SEPs Arriving at Mars Constrained by Remote Sensing and In Situ Measurement. *Space Weather* 2018; **16**: 1156–1169.
24. Dresing N, Gómez-Herrero R, Heber B *et al.* Statistical survey of widely spread out solar electron events observed with stereo and ace with special attention to anisotropies. *Astron. & Astrophys.* 2014; **567**: A27.
25. Roelof EC, Gold RE, Simnett GM *et al.* Low-energy solar electrons and ions observed at ulysses february-april, 1991: The inner heliosphere as a particle reservoir. *Geophysical Research Letters* 1992; **19**: 1243–1246.
